# A Retro-Perspective on Auxin Transport

**DOI:** 10.3389/fpls.2021.756968

**Published:** 2021-10-05

**Authors:** Markus M. Geisler

**Affiliations:** Department of Biology, University of Fribourg, Fribourg, Switzerland

**Keywords:** polar auxin transport (PAT), ABCB, AUX1/LAX, PIN, PILS, NPA, AGC kinase

## Introduction

The transport of the plant hormone auxin has been a hotspot in plant biology since its discovery (Darwin and Darwin, 1880; Zazimalova et al., 2010; Friml, 2021; Hammes et al., 2021). After its identification and verification as IAA (3-indolyl acetic acid; Went and Thimann, 1937), auxin gained high interest and fascination in the plant community but also in society because it allowed us to explain daily-seen phenomena, such as phototropism, gravitropism, patterning, and development (Christie and Murphy, 2013; Geisler et al., 2014; Morohashi et al., 2017; Konstantinova et al., 2021).

**Figure 1:**
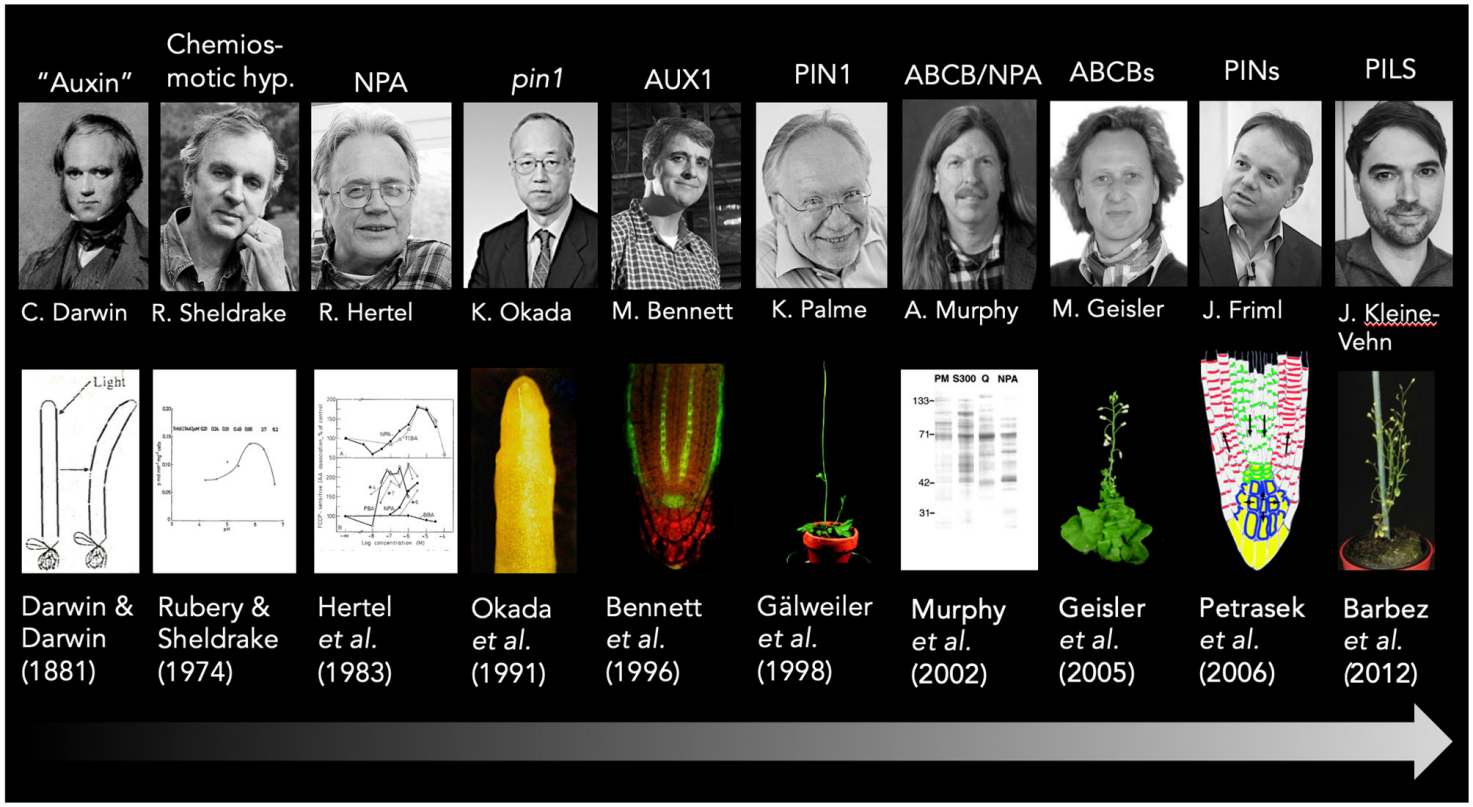
A short timeline of the auxin transport history. Key findings in auxin transport research that are discussed in this article are highlighted and correlated with the main responsible researcher and key publications; exemplary pictures are added for illustration purposes. Please note that due to space restrictions, some topics are only assigned to a single person, and acknowledge that major findings were conducted by several groups. This holds true for the formulation of the chemiosmotic hypothesis that was suggested independently by at least two groups Rubery and Sheldrake (1973), Raven (1975), and summarized later by Goldsmith (1977). Sincere apologies to all colleagues from the auxin transport field that contributed and are not included here. The *abcb1 abcb19* mutant picture is taken from Bailly et al. (2006), while the PIN reflux model is taken from Wabnik et al. (2011).

The mid twentieth century saw the emergence in the use of artificial and natural auxins as growth regulators and herbicides, and led to advances in reduced tillage agriculture as well as widespread military use of “auxinic” defoliants, such as 2,4-D (Friml and Palme, 2002). This first major wave of auxin research characterized by a predominantly biochemical characterization of auxin action in respect to growth lasted until the early 1990's and resulted in fascinating concepts, including the “chemiosmotic model of auxin transport” (Rubery and Sheldrake, 1973, 1974; Raven, 1975; Goldsmith, 1977; see Figure 1) and the “auxin canalization theory” (Sachs, 2000; Bennett et al., 2014; Ravichandran et al., 2020).

Then in the mid-1990's, the tools of molecular biology and the use of Arabidopsis as a model system provided the framework for breathtakingly rapid advancements that unwrapped many of the secrets underlying polar auxin transport and its role in plant development. This period allowed for the identification and characterization of multiple auxin transporter families (for details, see below) and the principal verification of the major theories. Excellent science led to a flow of beautiful publications that promoted auxin as the rising star of plant biology (Benjamins and Scheres, 2008).

If one undertakes a PUBMED key word search for “auxin transport”, the server returns more than 4.000 refereed publications from the period 1995–2021. Looking back, plant science in general has been influenced fundamentally by auxin transport research. At the same time, the community was also selling the fascinating cell-to-cell or polar transport of IAA as an auxin-specific and plant-unique phenomenon. However, as was the case with much research from the twentieth century, this period has not been without controversy, and some important publications from this period represent wrong turns that required retractions.

Under this light, this grand challenge article is not meant to provide an all-encompassing overview on auxin transport; for the interested reader, I refer to the many excellent reviews that have already been written and are cited below. Instead of providing another “lexicon of auxin transport,” the idea of this “retro-perspective” is to provide a brief overview on key aspects of auxin transport and use this opportunity to point out crucial misunderstandings and misconceptions, outline formal gaps and make concrete suggestions for urgent future work. The intention is to limit this article to a few arbitrarily selected aspects that are important for an understanding of the fascinating cell-to-cell or polar transport of IAA, the major native auxin.

## Polar Auxin Transport

In the 1960's, polar transport of radiolabeled auxin was definitively shown in pea stems and maize coleoptiles (Briggs, 1960; Leopold, 1964) and the hunt for the underlying mechanisms began in earnest. In the 1970's, auxin transport experiments combined with inhibitor studies (Rubery and Sheldrake, 1973, 1974; Katekar and Geissler, 1977) led to the formulation of the chemiosmotic hypothesis of auxin transport (Rubery and Sheldrake, 1973, 1974; Raven, 1975; Goldsmith, 1977) describing how IAA could move from cell to cell. Its basis is formed by the chemical nature of IAA, which as a weak acid (pK_a_ = 4.85) and can partially cross the plasma membrane from the apoplast (pH 5.5) but not from the neutral cytoplasm which requires an export system (Zazimalova et al., 2010). It was thus postulated that auxin is transported into and out of the cell through the action of specific carrier proteins (Rubery and Sheldrake, 1973, 1974; Raven, 1975; Goldsmith, 1977). It was also proposed that the strictly controlled directionality of auxin flow may be the result of an asymmetric cellular localization of auxin efflux carriers (Goldsmith, 1977; Martin et al., 1990).

However, several biochemical observations indicated that the simple concept of chemiosmotic auxin transport required further elaboration. In the apoplast at pH 5.5, only ~16% of IAA is protonated (Zazimalova et al., 2010). Studies in membrane vesicles and intact tissues predicted the presence of an auxin uptake symporter (Hertel et al., 1983; Lomax et al., 1985). The identification of ATPase activity and auxin binding sites on the plasma membrane predicted the presence of a vanadate-sensitive ATPase activity apart from the plasma membrane ATPases that contributed to auxin transport (Jacobs and Hertel, 1978; Jacobs and Taiz, 1980).

In the 1990's, the chemiosmotic hypothesis gained significant support from genetic and cell biology studies in *Arabidopsis thaliana* and led to the identification and characterization of auxin efflux and influx transporters of the plasma membrane belonging to the AUXIN-RESISTANT1/LIKE AUX1 (AUX1/LAX), the PIN-FORMED (PIN), and the B subgroup of ABC transporter (ABCB) families (Bennett et al., 1996; Galweiler et al., 1998; Luschnig et al., 1998). For the Arabidopsis root tip, a “reverse fountain model” was proposed based on transporter locations and mutant phenotype analyses in order to explain an auxin (signaling) maximum in the quiescent center (Swarup and Bennett, 2003). Computer models supported a self-sustaining “auxin reflux loop” that is thought to function as an “auxin capacitor” (Friml, 2003; Kepinski and Leyser, 2005; Benjamins and Scheres, 2008). These auxin reflux loops created by the combined action of multiple auxin transporters are thought to establish local auxin maxima and minima allowing auxin to act as versatile physiological and developmental switch (Vanneste and Friml, 2009). In that respect the mode of auxin action is eventually closer to a morphogen (Berleth and Sachs, 2001; Dubrovsky et al., 2008) rather than to a classical hormone.

**Grand Challenges:** The above outlined scenario defines transmembrane auxin transport over the plasma membrane as a major driving force for the establishment of local auxin gradients (Robert and Friml, 2009; Vanneste and Friml, 2009; Geisler et al., 2014). However, all steps in between, like apoplastic and cytoplasmic diffusion as well as vacuolar and ER compartmentalization, are still black boxes. While apoplastic IAA diffusion might simply follow a concentration-gradient provided by export and uptake systems, this might be slightly trickier for a cytoplasmic passage. This holds true especially for mature epidermal and cortical cells in the differentiation zone of the root tip, where the cytoplasm is limited to a small cytoplasmic strip. Also, it is unclear to what extent IAA metabolism, such as oxidation (Peer et al., 2013), and conjugation (Woodward and Bartel, 2005), as well as IAA compartmentalization into organelles (Sauer and Kleine-Vehn, 2019; Salazar-Iribe and De-la-Pena, 2020) has an effect on the polar auxin transport.

Connected to this, alternative concepts that are based on continuous, cytoplasmic auxin gradients over many cells that are inter-connected *via* a network of plasmodesmata might be worth considering. In such a model, auxin transporters would solely provide local auxin sinks at plasma membrane subdomains needed for cytoplasmic diffusion. As a support of such a highly speculative concept, recently several studies have revealed that plasmodesmata contribute to auxin distribution, and that a manipulation of these transport pathways alters auxin-related phenotypes (Band, 2021; Winnicki et al., 2021).

Another relevant question arises, is such a cell-to-cell delivery limited to auxin? In fact, there are now multiple reports on the transport of other hormones, such as cytokinins, abscisic acid (ABA) and gibberellic acid (GA), where short-distance delivery for the creation of gradients have been described (Geisler, 2018; Anfang and Shani, 2021). Obviously, for ABA (p*K*_a_ = 4.74) and GA (p*K*_a_ = 4.04), which are also weak acids, cellular compartmentalization dependent on pH can be assumed.

Another grand challenge is to view auxin transport in real-time. Real-time imaging of auxin flows has been limited by the absence of a dynamic auxin sensor because transcriptional and degron-based auxin reporters are excellent tools, but simply too slow to allow for *in vivo* imaging of auxin transport in real time (Geisler, 2018; Walia et al., 2018). With the recent development of the FRET-based auxin sensor, AuxSen (Herud-Sikimic et al., 2021), the auxin transport field has no excuses anymore to tackle these questions. A major advantage of AuxSen is the usage of heterologous proteins as binding domains (here: the bacterial Trp repressor) over plant endogenous proteins because they are unlikely to interfere with plant signaling pathways. In the case where a sole auxin sensor with a fixed IAA affinity is not suitable to report auxin gradients over several cell files with different local auxin concentrations, multiparametric imaging could be achieved by expressing multiple auxin sensors. Recently, a 2-in-1 genetically encoded fluorescence indicator fused *via* a 14-amino-acid linker was established (Waadt et al., 2020). In addition, these auxin sensors might be combined with auxin transport activity sensors (Isoda et al., 2021) that could provide an indirect read-out for auxin transport. Pioneering work in animal systems has enabled the multiplexing and simultaneous recording of many processes, in part through differential subcellular targeting and in part through the development of fluorescence-lifetime imaging (FLIM) sensors (Greenwald et al., 2018; Isoda et al., 2021).

## Auxin Transporters

In light of the fact that auxin, and thus also auxin transport, is involved directly or indirectly in so many if not all physiological and developmental processes in higher plants, it is not that surprising that plants have recruited a plethora of auxin transporters from different transport families (Zazimalova et al., 2010). Currently, the four main auxin transporter families are comprised of the AUX1/LAX (with 4 isoforms in Arabidopsis), the PIN (8), the ABCB (11), and the PIN-LIKES (PILS; 7) families [reviewed in Zazimalova et al. (2010) and Hammes et al. (2021)]. While most members of the former three families reside as expected on the plasma membrane, short PIN (Mravec et al., 2009; Ding et al., 2012) and PILS proteins (Barbez et al., 2012) are found predominantly at the ER, where they contribute primarily to auxin homeostasis (Schwuchow et al., 2001; Barbez and Kleine-Vehn, 2013). AUX1/LAX isoforms were shown to function as importers (Yang et al., 2006), long PINs are thought to export auxin, while ABCBs mainly export, however, import directionalities were also reported (Geisler et al., 2005; Santelia et al., 2005; Terasaka et al., 2005; Kamimoto et al., 2012; Ofori et al., 2018; Zhang et al., 2018).

ABCBs were initially a challenge for the auxin transport community because their substrate specificity was equated with human ABCB-type multi-drug transporters. However, transport experiments demonstrated that the plant transporters exhibited a remarkable specificity to auxin (Geisler et al., 2005). Moreover, unlike for PIN, AUX1/LAX, and PILS proteins, not all ABCB isoforms are auxin transporters (Park et al., 2017; Ogasawara et al., 2020). Furthermore, a gene duplication event in the ABC transporter family (Ogasawara et al., 2020) hindered their identification in classical genetic screens (Zhang et al., 2018). In between, based on the identification of a signature D/E-P motif for auxin transporting ABCBs (ATAs) it was suggested that 11 of the 22 full-size ABCBs are ATAs (Geisler and Hegedus, 2020; Hao et al., 2020). In between, functional redundancy between similar ATA isoforms could be solved by using clade-specific gene silencing (Zhang et al., 2018).

Interestingly, AUX1, PIN1, and ABCB1/PGP1 were already identified in the mid 1990's (Bennett et al., 1996; Galweiler et al., 1998; Sidler et al., 1998; see Figure 1), however, it took nearly another decade until their auxin transport activities were verified by whole-cell transport studies (Geisler et al., 2005; Petrasek et al., 2006; Yang et al., 2006). Today it is clear that the choice of whole-cell assays enabled confident measuring of auxin transport by reducing IAA diffusion due to a more favorable surface-to-volume ratio compared to smaller microsomal vesicles. A major drawback of whole-cell export assays is that it only permits a semi-quantitative analysis of export capacities because they require an uncontrolled loading step. The assay only offers measuring uptake kinetics for importers as shown for AUX1 (Yang et al., 2006).

Beside these four major classes of transporter, there is an increasing number of new putative auxin transporters from other transporter families that, based on the fact that they were originally assigned to other substrates, were recently called “moonlighting” auxin transporters (Hammes et al., 2021). In my view, this assignment is not fully correct because *moonlighting proteins* are defined by a second, unrelated function. The transporters NRT1.1/ NPF6.3/ CHL1 (Beeckman and Friml, 2010; Krouk et al., 2010; Wang et al., 2020) or WAT1/UmamiT5 (Ranocha et al., 2013) are more likely to have dual (or multiple) substrate specificities. For most of these transporters, despite having convincing auxin-related phenotype, clear-cut auxin transport activity awaits confirmation.

An excellent review on auxin transporters has raised the slightly provocative question “Auxin transporters—Why so many?” (Zazimalova et al., 2010). At the time only 15 auxin transporters were described in Arabidopsis. The community is now confronted with a minimum of 30 Arabidopsis auxin transporters from three major families that are all energized differently. The generally accepted chemiosmotic model of auxin transport initially pointed to AUX1/LAX proteins rather than to PINs and ABCBs, which are driven by electrochemical gradients and ATP hydrolyses, respectively. However, the same transporter profile is also found in other essential signaling molecules, such as the secondary messenger Ca^2+^, that employs calcium channels, Ca^2+^/H^+^ antiporters, and Ca^2+^ATPases of the P-type (Geisler et al., 2000). In principle the same *modi operandi* are used by PINs/PILSs, AUX1/LAXs, and ABCBs where auxin is moved by electro-chemical gradients, H^+^ symport or ATP hydrolysis. As it stands, evolution apparently favored the availability of multiple, energetically distinct transport systems for essential signaling molecules.

On the other hand, the high number of auxin transporters might not come as a big surprise because the over-representation of transport systems is a general plant strategy and is considered an adaptation to its sessile life style (Kang et al., 2011; Kretzschmar et al., 2011; Park et al., 2017; Anfang and Shani, 2021). Also, the *chemiosmotic model* “might have gotten something wrong” by predicting mainly auxin exporters on the plant plasma membrane: in Arabidopsis roughly half (14 out of 30) of the auxin transporters are plasma membrane exporters, while six out of the 30 are cellular importers, while 10 are internal importers. These simple numbers might suggest that the role of auxin uptake and homeostasis for plant performance are slightly underestimated.

**Grand Challenges:** In the near future, we urgently need a thorough biochemical characterization of key auxin transporters to enable us to assign their role in PAT. As explained above this is currently hindered by the use of whole-cell transport systems as a concession toward IAA diffusion. A way forward could be to use synthetic auxin analogs that ideally had similar kinetic properties but reduced diffusion rates.

Connected to this, another important milestone is to investigate the suggested interplay between auxin transporters, such as PIN-ABCB pairs (Bandyopadhyay et al., 2007; Blakeslee et al., 2007; Mravec et al., 2008; Teale et al., 2021). Previous studies point to a functional interaction between these transporter classes influencing transport capacities, directionalities and inhibitor sensitivities (see below; Blakeslee et al., 2007). However, this exciting concept is far from being understood and was indirectly questioned recently (Teale et al., 2021). The way forward is probably difficult and would require protein purification and reconstitution in a cell-free system.

As a spin-off from this protein work, structure-function analyses should be envisaged for key members of all auxin transporter families. Remarkably, no crystal or cryo-EM structure of any auxin transporter exists, and considering its importance as a signaling molecule, slightly embarrassing for the auxin community. Symporter and ABCB structures from different non-plant sources are available and it would be informative to assess structural differences to non-auxin transporting orthologs. Of special interest are evolutionary conserved differences in putative substrate (auxin) binding domains, which could be easily identified by co-crystallization. Of importance are also PIN protein structures, less in respect to their transport mechanisms but in their regulation by loop phosphorylation (Hammes et al., 2021). One should also not forget that, in contrast to ABCBs and AUX1/LAX proteins, PINs form a plant-specific subgroup of MFS transporters, and therefore a structure would be of special interest.

In principle, all transporter locations align well with known auxin streams in the root tip and mutant phenotypes in Arabidopsis, however, one should not forget that the latter were also mainly deduced from transporter expression, which is only a very indirect proxy for substrate streams at best (Geisler, 2018). However, assignment of a specific auxin transporters in this complicated auxin transport network at the plant level seems to have reached its limitations through the use of classical genetics and biochemistry. The reason lies in the redundant and the interactive action of the many auxin transporters from different families. Another level of complication is added by the fact that we are facing a mobile signal. Therefore, the successful methods of first defining auxin transport streams and then to assign transporters to these streams (Kuhlemeier, 2007) may have reached its limitation. An alternative route is offered by mathematical modeling, which can integrate multiple transporters from different transporter families (Kramer, 2008; Band et al., 2014; Middleton et al., 2018). This has already been done for PIN export and AUX1/LAX influx carriers, respectively (Band et al., 2014; Middleton et al., 2018). Especially convincing was a recent combination of mathematical modeling that included PIN locations and auxin maxima deduced from experimental (confocal) data (Band et al., 2014). An extension of such work on ABCBs and even a combination of transporters from distinct transporter families should be very informative. Such studies should also include the different turnover numbers for transporters of the different subclasses; currently they are considered to transport equally. The advantage is that *via* mathematical modeling a high number of transporters and transporter combinations can be tried, this would enable testing a near unlimited number of hypotheses.

## Auxin Transporter Evolution

The recent evo-devo (evolutionary developmental biology) wave has not stopped at auxin either (Friedman, 2009; Finet and Jaillais, 2012; O'Connor et al., 2017). While evolutionary analyses on nuclear auxin signaling components have been done (Kato et al., 2018; Blazquez et al., 2020), this unfortunately cannot be said for auxin transporters. This is a pity because sequence and expression data covering lower plants and algae are becoming publicly available and would allow some urgent questions to be addressed (outlined above). For example, the identification of “old” transporter families in an evolutionary sense and the assignment of other transporter classes to key developmental innovations would allow us to make predictions on the origin of auxin transport and at the same time to assign specific roles to these transporter families. On the other hand, such analyses have been hampered by the fact that, unlike for other transporters or auxin signaling components, it is “nearly” impossible to confidently predict auxin transport specificity simply by sequence homology. This is especially the case in this type of analysis as homology decreases drastically with phylogenetic distances.

Only a few studies using different approaches at different quality levels have addressed auxin transporter evolution so far and those have limited their attention to the green lineage (*Viridiplantae*) comprising chlorophytes and streptophytes (Viaene et al., 2014; Skokan et al., 2019; Zhang et al., 2019; Vosolsobe et al., 2020). The unified current picture that emerges is that ABCBs (virtually found in all domains of life) and PILS are ancient auxin transporters, while PINs and AUX1/LAXs are more recent lineages (Vosolsobe et al., 2020). Despite being found in most charophytes, PINs can be less frequently identified in chlorophytes (Vosolsobe et al., 2020). The different origins of PIN and PILS proteins is somewhat surprising as PILS were originally identified based on sequence homology (Barbez et al., 2012) and as such both contain a diagnostic *Auxin efflux carrier component 2* (IPR033526) motif. However, recently good evidence for an independent evolution was provided (Feraru et al., 2012). The origin of AUX1/LAX transporters showing a fragmentary distribution over charophytes and chlorophytes (Vosolsobe et al., 2020) is less clear.

A recent thorough analysis (Vosolsobe et al., 2020) pointed out several remarkable surprises: First, in some basal charophytes, such as *Chlorokybophyceae*, all secondary auxin transporters (PIN, AUX1/LAX, and PILS) are secondarily lost, meaning that these algae mainly rely on ABCBs. Second, the most complex algae, *Chara*, showing a nearly plant-like stature and clear evidence for PAT, contains only PIN and ABCB-type auxin transporters.

In summary, it appears that all four transporter classes have evolved independently and are usually not present in any single algae, with the exception of *Klebsormidium sp*. (Vosolsobe et al., 2020). The previous view that PINs have arisen with the presence of a vasculature and thus with the water-land-transition is apparently not true (Galvan-Ampudia and Offringa, 2007; Vosolsobe et al., 2020). This does not exclude that PINs, generally thought to provide a high degree of developmental plasticity, might be needed for the newly established sessile lifestyle where new physiological requirements (such as gravitropism and phototropism) play an important role (Bennett, 2015). But this role of PINs is most likely attributed to their diversification in land plants (Bennett, 2015). However, in this context it should also be kept in mind that gravitropism is not a strict requirement for the establishment of auxin gradients as they are known to exist in space (Ferl and Paul, 2016). In light of these findings, the previous concept that ER-based auxin homeostasis instead of plasma membrane export is the ancient auxin transport system (Viaene et al., 2013) is probably off the table. Finally, despite original predictions that auxin transporter polarity seems to be a newly acquired it is not essential for PAT.

**Grand Challenges:** In the next few years, the community urgently needs to enhance our knowledge on auxin transporter evolution because this might offer an understanding of auxin action as a signaling molecule *per se*. An interesting venue may be provided by understanding why unicellular organisms, such as green algae, need an auxin export system at all. This may originally have represented an excretion system liberating the cells of toxic by-products of metabolism [like in mammalian tumor cells or during some human diseases (Chanclud and Lacombe, 2017)] or allow the cells to export IAA as a signaling molecule allowing for intercellular communication during intraspecific quorum sensing (Chanclud and Lacombe, 2017; Vosolsobe et al., 2020). Another idea is that in unicellular organisms there might a need for auxin gradients permitting physiological reactions, such as growth promotion. For the unicellular moss, *Ceratodon purpureus*, it was shown that disruption of auxin export by NPA interferes with unicellular gravitropism of the protonema (Schwuchow et al., 2001).

In order to do so, we need more genomes from under-represented algae lineages and evolutionary analyses need to be carried out more thoroughly, like done for the auxin signaling components (Blazquez et al., 2020). Analyses based on sequence homology that include key elements defining substrate specificity or regulation [such as the D/E-P motif for ABCBs (Hao et al., 2020) or the *Auxin efflux carrier component 2* (IPR033526) motif for PINs and PILS (Feraru et al., 2012)] might be the way to go. While current analyses have focused for good reason around the water-land transition and thus on the green lineage, this scrutiny must urgently be extended to other algae and non-Arabidopsis plants, especially crop plants. Of special interest will be brown algae for that developmental effects caused by IAA are reported (Bogaert et al., 2019).

A further grand challenge is the co-evolutionary analysis of auxin transporters and regulatory components, such as kinases and chaperones. This has been partially initiated for PINs and members of the AGC kinase family that seem to have co-evolved (Galvan-Ampudia and Offringa, 2007). Such an analysis is of interest because prominent members of this family, such as PINOID and phot1, were also shown to regulate ABCB transport activity (Christie et al., 2011; Henrichs et al., 2012; Christie and Murphy, 2013), which would suggest that these functional interactions were acquired secondarily.

In any case, it will be essential to tie-up any conclusion from evolutionary analyses of transport studies to prove predicted auxin transport activities and substrate specificities. This is because homology-based predictions have their pitfalls. Additionally, such transport studies should be confirmed through functional complementation of auxin transporter mutants in Arabidopsis as has been recently started for ancient PIN isoforms (Skokan et al., 2019). Interestingly, the most primitive *PIN* gene known to date from the basal *Streptophyte* green alga *Klebsormidium flaccidum* was unable to rescue the defects in root gravitropism in the *pin2* mutant (Zhang et al., 2019), although it was shown to be a functional auxin transporter (Skokan et al., 2019). Finally, there is an urgent need to establish algal models to enable direct auxin transport measuring and genetic access.

## Auxin Transporter Regulation

As can be expected for an essential signaling molecule, like auxin, its transmembrane distribution by auxin transporter proteins is tightly regulated at the transcriptional and post-transcriptional level (Benjamins et al., 2005; Robert and Offringa, 2008; Geisler et al., 2016, 2017; Hammes et al., 2021). Over the last decades for the different transporter families, different depths of understanding toward their regulation have been provided but it is probably safe to predict that auxin transporters (like most other transporters) are regulated at all known aspects of post-transcriptional regulation, including transport activity, membrane trafficking, and protein stability.

For a long time, the auxin community mainly focused on the establishment and maintenance of transporter polarity, with a special emphasis on the trafficking routes of PIN proteins (Rakusova et al., 2015; Zhou and Luo, 2018; Han et al., 2021). In short, PINs are constitutively internalized on clathrin-coated vesicles (Kleine-Vehn and Friml, 2008; Kleine-Vehn et al., 2008) and recycled back to the plasma membrane. These processes are regulated by a wealth of regulatory factors, including various GTPases, ARF-GEFs, and ARF-GAPs (Chen and Friml, 2014; Adamowski and Friml, 2015; Friml, 2021; Han et al., 2021). Another regulatory module orchestrating PIN polarity is formed by the interplay of AGC kinases and protein phosphatase 2A which regulate the phosphorylation status of cytoplasmic PIN loops (for details, see below; Michniewicz et al., 2007; Robert and Offringa, 2008; Huang et al., 2010; Offringa and Huang, 2013). This focus drove the prediction of the chemiosmotic model but also integrated auxin transporter networks into the main physiological read-outs of root gravitropism and shoot phototropism. On the other hand, for many years advances in PIN biochemistry were stuck because all attempts to demonstrate auxin transport for PIN proteins failed due to technical reasons.

As of today, a key concept is promoted that is partially based on the chemiosmotic model. This concept emphasizes transporter polarity as the basis for the polar distribution of auxin (Wisniewska et al., 2006), however this has not yet been verified. Along the same lines, dynamic transporter cycling has been suggested as a strict requirement for transporter polarities and both criteria together have served as a benchmark for auxin transporters. Thus, a central question for the future is to what extent is transporter polarity (and transporter dynamics) a requirement for polar transport. This is important as any uniformly, localized transporter can be activated on polar subdomains by local regulatory events, like protein phosphorylation (Christie and Murphy, 2013).

This brings us to a developing field that has demonstrated that auxin transport depends on the activity of a subgroup of plant-specific serine/threonine kinases, the so called AGC kinases (Galvan-Ampudia and Offringa, 2007; Rademacher and Offringa, 2012). Members of the AGC kinase subclade VIII were shown to phosphorylate PINs and ABCBs on their cytoplasmic loops leading to activation of long PINs (Zourelidou et al., 2009, 2014; Barbosa and Schwechheimer, 2014; Hammes et al., 2021). For ABCB1 and ABCB19, the activating and inhibiting effects on auxin transport by the AGCVIII kinases (PINOID and phot1), result in defects in gravitropism and phototropism (Christie et al., 2011; Henrichs et al., 2012; Christie and Murphy, 2013). Opposite effects on ABCBs by AGC kinase phosphorylation were discussed to be caused by interaction between the ABCBs and the immunophilin-like FKBP42, Twisted Dwarf1 (TWD1), which is thought to recruit the AGC kinases (Christie and Murphy, 2013; Geisler et al., 2016). Overall, the developmental phenotypes reported for AGCVIII kinase mutants align well with those of the respective kinase substrate mutants, which is probably best illustrated by the phenotypes of the *pinoid* and the *pin1* mutants, showing overlapping degrees of pin-shaped inflorescences (Benjamins et al., 2001; Friml et al., 2004).

An interesting finding is that some kinases of the AGC3 and AGC4 subcluster, such as PINOID and phot1, phosphorylate auxin transporters from different subclasses, like PIN1/ABCB1 and PIN3/ABCB19, respectively (Geisler et al., 2016). Remarkably and also puzzling is that AGC1 and AGC3 kinases target the same phosphorylation sites of PIN proteins but that AGC3 kinases (unlike AGC1 kinases) were initially found to regulate PIN polarity (Hammes et al., 2021). This has caused debates in the community mainly because the two major “factions” insisted on a exclusivity claim for their findings, while widely ignoring the option that both are not mutually exclusive. Indeed, a clean dissection of both events is technically challenging because both an increase of transporter polarity and transporter activity would lead to enhanced transport, which in the context of auxin canalization would be self-amplifying.

Finally, auxin transporter folding by PPIases (*cis-trans* peptidylprolyl isomerases) seem to have both an effect on PIN and ABCB transport activity and trafficking (Geisler and Bailly, 2007; Geisler et al., 2016). TWD1 was shown to function as chaperone during early ABCB biogenesis based on the finding that ABCB1,4,19, unlike PINs, are retained and degraded at the ER in the *twd1* mutant (Wu et al., 2010; Wang et al., 2013). As a result, *abcb1 abcb19* plants resemble the *twd1* mutant and show similar PAT defects (Geisler et al., 2003; see Figure 1). However, auxin-transporting ABCBs (ATAs) contain an essential proline as part of a diagnostic D/E-P motif in their C-terminal nucleotide-binding folds that is essential for auxin transport but not for trafficking (Geisler and Hegedus, 2020; Hao et al., 2020). Thus, TWD1 might have a dual role in ABCB activation and secretion, respectively, which is an analogy to human FKBP38 (Geisler and Hegedus, 2020).

Similarly, PIN1 was shown to be folded and regulated by the parvulin, PIN1At, known to fold proline residues preceding phosphorylation sites (Xi et al., 2016). However, it is not entirely clear if these events lead to altered transport activity or transporter polarity or both. ABCB1 contains a series of prolines in the vicinity of putative phosphorylation sites in its regulatory linker (Henrichs et al., 2012), however it is unknown if folding and phosphorylation events are interconnected.

**Grand Challenges:** Future grand challenges include a proper dissection of regulatory events on auxin transporters from different classes. This is indeed important because it is currently not yet clear whether protein phosphorylation leads to transport activation on the transport or polarity level. Moreover, a thorough investigation of overlapping kinase activities on members of different transporter subclasses is essential. Both can be addressed by *in vitro* and *in vivo* biochemistry (Jones et al., 2013; Geisler, 2018). The latter requires an integration of regulatory components, like kinases, chaperones, etc., and their effect on protein stabilities and transport activities, which will allow for a prediction of fluxes over time. The techniques to image kinase or transporter activities (by usage of transport activity sensor and SPARK (Separation of Phases-based Activity Reporter of Kinase) assays) and transporter-regulator interaction (by using FRET) are available and need to be transferred or optimized to the plant field (Geisler, 2018).

## NPA

As for other disciplines, the identification of pharmacological inhibitors was extremely helpful for auxin transport research. In the late 1950's, a series of phtalamic acid derivates were reported to inhibit tropic bending, coining the name *phytotropin* (Morgan and Söding, 1958). Since the work in the 1980's on maize coleoptile segments (Hertel and Flory, 1968) and vesicles (Hertel et al., 1983), we know that NPA is a non-competitive inhibitor of auxin efflux but not of growth. What is less recognized is that NPA differentially inhibits the export of IAA and synthetic auxin, such as 1-NAA and 2.4-D (Delbarre et al., 1996). Also overlooked is that NPA, like other phytotropins, is thought to bind to the same receptor, through which it performs its physiological responses (Katekar and Geissler, 1975; Geissler et al., 1985; Michalke et al., 1992). This has led to speculation that the exporter might own a transceptor-like function (Hossel et al., 2005).

In the 1990's, different groups invested an enormous effort in characterizing the number, affinities and identities of putative plasma membrane-based NPA targets (Michalke et al., 1992; Cox and Muday, 1994; Bernasconi et al., 1996; Dixon et al., 1996; Butler et al., 1998; Teale and Palme, 2018). The overall outcome as reviewed in (Teale and Palme, 2018) revealed a very complex, partially contradicting picture with respect to the number and nature of the targets (Teale and Palme, 2018).

A route to the identification of an NPA target was provided by the isolation of the mutant allele *pin-formed1* (*pin1*) that resembles plants grown on NPA (Okada et al., 1991; see Figure 1). Consecutively, the *PIN1* gene was cloned and PIN1 was identified as a member of the major facilitator superfamily with a striking polar localization (Galweiler et al., 1998). This correlation served as a quasi-accepted proof that PINs in general are NPA-sensitive auxin exporters, which was finally demonstrated in tobacco BY2 cells (Petrasek et al., 2006). For some time, a puzzling finding for the community was that PIN1 was inactive in heterologous non-plant systems, such as yeast or oocytes, shedding some doubt on its direct function as a transporter. However, also this missing detail was solved by the finding that PIN1-mediated transport is strictly dependent on phosphorylation, which was provided either by AGC kinase co-expression or phospho-mimicry (Zourelidou et al., 2009; Wang et al., 2012). Recently, two independent reports using oocytes and Arabidopsis protoplasts further validated PINs as direct targets of NPA (Abas et al., 2021; Hammes et al., 2021; Teale et al., 2021). Interestingly, one provided evidence that PIN1 inhibition by NPA does not involve classical allosteric inhibition but acts *via* an induction of PIN homo- and heterodimers, which is counteracted by PIN1 phosphorylation and IAA (Teale et al., 2021).

Another line of NPA inhibition of auxin exporters was developed by the identification of ABCB transporters and TWD1 by NPA-affinity chromatography (Noh et al., 2001; Murphy et al., 2002). Consequently, ABCBs and TWD1 were confirmed to bind NPA (Geisler et al., 2003; Kim et al., 2010; Zhu et al., 2016) and ABCB-mediated export was found to be NPA-sensitive (Geisler et al., 2005; Bouchard et al., 2006; Kim et al., 2010). NPA, like different flavonols, was able to disrupt ABCB-TWD1 interaction suggesting that NPA might bind at their interface (Bailly et al., 2008). During this time, the NPA binding site on the so-called FK506-binding domain (FKBD) of TWD1 had been precisely mapped by NMR and chemical density mapping was verified by mutational analyses (Zhu et al., 2016). Based on *in silico* docking and again verified by mutagenesis, NPA is thought to bind to the C-terminal nucleotide binding fold (NBD2) of ABCB1 (Kim et al., 2010), which is both in agreement with the finding that the interaction is provided by FKBD and NBD2 of TWD1 and ABCB1, respectively (Geisler et al., 2003).

Using chemical-genetic screens, the NPA analog, BUM (2-[4-(diethylamino)-2-hydroxybenzoyl]benzoic acid), was identified and shown to have an IC_50_ value that is roughly a factor 30 lower (Kim et al., 2010). Physiological analysis and binding assays identified ABCBs, primarily ABCB1, as key targets of BUM, whereas PIN proteins were shown to be not be directly affected (Kim et al., 2010). TWD1 seems to own a second function on auxin transport that involves bundling of the actin cytoskeleton (Zhu and Geisler, 2015; Zhu et al., 2016). TWD1 is required for NPA-mediated actin remodeling that seems to involve ACTIN7, which itself is responsible for proper plasma membrane trafficking of PINs and ABCBs (Zhu and Geisler, 2015; Zhu et al., 2016). Interestingly, both the epidermal twisting in *abcb1 abcb19* and *twd1* can be partially rescued by NPA treatments (Wang et al., 2013), indicating that NPA targets beside TWD1 and ABCBs might be involved. Another promising outcome of the initial NPA-affinity chromatography (Murphy et al., 2000, 2002) was the aminopeptidase, APM1, that was characterized as a low-affinity NPA-binding protein. The *apm1* mutant has reduced PAT and PIN and ABCB delocalization (Peer et al., 2009).

**Grand Challenges**: Overall it seems that 60 years after its first description, we now have a slightly better understanding of NPA action and it is good to see that initial predictions that NPA interferes primarily with the efflux complex seem to hold true. It is now clear that the path to understand NPA was heavily complicated by the fact that there are multiple NPA targets in plants, each with different binding affinities that partially interact with each other. On top it was shown that some of these interactions, such as between PINs and ABCBs, can influence the binding affinities of these complexes (Blakeslee et al., 2007). Another level of complication is caused by the fact that NPA seems to interfere with transporter phosphorylation. This is highlighted by the finding that the protein phosphatase subunit 2A, called *Roots Curl under NPA1* (*RCN1*), a regulator of PIN transcytosis (Michniewicz et al., 2007), was identified in chemical genetic screens under NPA (Garbers et al., 1996; Deruere et al., 1999). Further, NPA was also suspected to alter auto-phosphorylation of PINOID by direct binding (Henrichs et al., 2012). Finally, there are reports that NPA might directly interfere with actin bundling in an action that is independent of TWD1 (Dhonukshe et al., 2008; Zhu and Geisler, 2015), which could alter auxin transporter trafficking directly.

It is remarkable that our understanding of the mechanism of such an important research tool used in so many labs around the world is still incomplete. Priority must be given to the biochemical characterization of NPA binding sites on known targets (such as PINs and ABCBs) by NMR, SPR (or similar), and NPA co-crystallization. At the next level, a systematic *in planta* dissection of NPA-sensitivities of auxin transport complexes must be achieved using suitable approaches, such as quantitative proximity ligation assays (PLA; Teale et al., 2021). Having the protein targets in hand, would allow for the development of specific (efflux) inhibitors that are more selective toward a certain transporter class.

Moreover, it will be essential to completely understand the overlapping *pin-formed* phenotype that is thought to be caused by genetic (*pin1, pinoid*) or pharmacological inhibition (NPA, BUM) of PAT that has branded the *PIN* subfamily. Despite our progress, it is still noteworthy that until today a plausible explanation for the inflorescence defects in *pin1* is still missing, especially in light of the fact that auxin levels in these tissues are not different to wild-type (Jones et al., 2005). Furthermore, one should not forget that growth on NPA (or BUM) likely leads to a saturated inhibition of all NPA targets in the plant making pin-formed inflorescences most-likely a pleiotropic phenotype. The finding that such as a phenotype is copied by single *pin1* or *pid* mutations suggests that PIN1 and/or PID most likely interfere with an overlapping subset of multiple downstream targets. That PIN1 was recently found to form complexes with multiple proteins, including other PIN isoforms, supports this overall concept (Blakeslee et al., 2007; Teale et al., 2021).

Finally, a continuously open question is the existence of a native NPA analog, which was originally assigned to flavonol derivates based on their ability to compete out NPA in binding assays and their ability to inhibit PAT (Murphy et al., 2000; Brown et al., 2001; Peer et al., 2001; Teale and Palme, 2018). For a while these were discarded (Peer and Murphy, 2007; Teale and Palme, 2018), however, recent work showing that they inhibit PIN transport by dimerization in analogy to NPA might place them back on the table (Teale et al., 2021). However, in this respect it might be important to recall that this effect (like the one for NPA) could be also simply caused by inhibition of kinases involved in PIN phosphorylation that would lead to a similar result.

## The Real Grand Challenge

In the last few years, the auxin transport community wasted a lot of energy on discussions about which auxin transporter family or regulatory component or concept is more “important” for auxin transport. While the usage of “importance” is a rather volatile term in evolution, the criteria for such a ranking were remarkably unscientific, being more personal and arbitrary in nature. In a trial to promote their “own” family or concept of auxin transport, simplistic and generalist assignments were created that sometimes did not reflect the truth and lacked experimental proof. These ideas persist today in the community and are thus very difficult to revise.

This created an atmosphere that was built on doubt and ignorance, and did not promote scientific progress. In that respect, I would like to suggest a reset and that we should become again interested in differences between auxin transporters with respect to their polarity, their mode of energization, plasma membrane stability or NPA sensitivity. We should see differences in auxin transport data more like a challenge than a flaw, which is in general probably a good mindset.

Throughout this perspective article, I have summarized and critically evaluated current knowledge as well as the many inconsistencies in the field. I have considered what could be done if energies and resources were fostered. In my eyes, the perspectives are clear but will require a better and more neutral, meaning a less self-centered, approach. Such a change in attitude might represent the biggest future challenge for the community. But it is worth trying as it has the potential to assist us to refocus on the essentials, which is after all the beauty of auxin transport. As a positive, it will help us to regain lost trust inside the plant community.

In addition to the grand challenges for basic research of auxin transport, we urgently need to better integrate with the applied sciences. Considering the important roles that auxin transport plays for plant development, we should keep an eye to the future of life on the planet. This focus might include the production of food, forage, fiber, fuel and pharmaceuticals as well as ecosystem services. We need to apply our basic research to societal questions, like feeding our children's children, environmental questions, like growing plants in climates where we already see changes that negatively impact quantity and quality of plant products and species diversity.

## Author Contributions

MG conceptualized and wrote the article.

## Funding

This work was supported by grants from the Swiss National Funds (31003A-165877/1).

## Conflict of Interest

The author declares that the research was conducted in the absence of any commercial or financial relationships that could be construed as a potential conflict of interest.

## Publisher's Note

All claims expressed in this article are solely those of the authors and do not necessarily represent those of their affiliated organizations, or those of the publisher, the editors and the reviewers. Any product that may be evaluated in this article, or claim that may be made by its manufacturer, is not guaranteed or endorsed by the publisher.
